# Spina Bifida

**Published:** 1844-02-24

**Authors:** 


### SPINA BIFIDA.

At a meeting of the Surgical Society of Ireland, Dr. Mitchell exhibited a specimen of this malformation, and stated that "the person who gave birth to it had been first seen by one of the pupils of the South-Eastern Lying-in Hospital, and when he first saw the fœtus he took it for a case of spina bifida anterior, from the strong resemblance which it bore to Cruveilhier's specimen of that affection. It proved, however, to be an extraordinarily large specimen of spina bifida posterior, situated in a very unusual place, inasmuch as that it extended from the third cervical to the fifth dorsal vertebra, the spinous process of all the intermediate vertebræ being deficient. The bones of the head had been also arrested in their development, and their positions much altered—the petrous portion of the temporal bone occupying the situation of the occipital. The brain was enclosed in a cyst, having a division down the centre, and communicating with a sac that covered the spine. The thoracic viscera were all natural, with the exception of the heart, which was of a disproportionate size. The abdominal viscera were quite normal; and one of the most striking facts in connexion with the monster was, that the platysma myoides was developed to a most extraordinary degree. The creature lived for about twenty minutes, and from the circumstance of the eyes being very prominent—he might say protruding—and there being a remarkable projection of the upper jaw, leaving the sacs of the teeth fully exposed, it presented a most hideous aspect—so much so, that it considerably frightened the nurse in attendance. It was not his intention to enter on the question of the formation of such monsters, or in what way the mind of the patient could act upon the fœtus in utero under powerful impressions. He would merely observe, that the woman attributed the cause of her having that malformation to the fact of a cat having been thrown on her back when about two months pregnant."

In the discussion which followed on the influence of external circumstances on the fœtus in utero, Dr. Houston, related "the case of the lady of a dispensary surgeon who was still living. Her husband had been engaged one day in an operation on a man who had received an injury on one of his hands by which either two or three of his fingers had been chopped off. While in the act of dressing the man's hand, his lady happened to have some message to deliver to him, and looked into the room, when she saw the bloody hand with the fingers wanting. She was at the time in an early stage of pregnancy, and when delivered, her child was born with the corresponding fingers missing, which the individual referred to had lost. The lady had had children previously who were all regularly formed; but every child she has since had has been born with the same defect. The case made a strong impression on the mind, that mental affections of the mother, while *enciente*, produced an effect on the fœtus in the womb."—*Dub. Med. Press*, Dec. 27.

